# Individual and joint effects of exposure to phthalates and the risk of cardiovascular disease in the chronic kidney disease population: NHANES 2005–2018

**DOI:** 10.3389/fpubh.2025.1579618

**Published:** 2025-05-14

**Authors:** Yuehong Liang, Xiurong Li

**Affiliations:** Department of Nephrology, Women’s Hospital of Nanjing Medical University, Nanjing Maternity and Child Health Care Hospital, Nanjing, China

**Keywords:** phthalates, cardiovascular disease, chronic kidney disease, NHANES, cross-sectional study

## Abstract

**Background:**

There is a lack of studies on the relationship between urinary phthalate metabolites (UPMs) and cardiovascular disease (CVD) prevalence in adults suffering from chronic kidney disease (CKD). We intended to examine the relationship between UPMs and the prevalence of CVD in people with CKD.

**Methods:**

The research utilized data in the National Health and Nutrition Examination Survey (NHANES) 2005–2018. We employed three statistical models—a generalized linear regression model, a weighted quantile sum (WQS) regression model, and a Bayesian kernel machine regression (BKMR) model.

**Results:**

We included 834 CKD participants finally. In the generalized linear regression model, the prevalence of CVD was higher in individuals with MiBP (OR 1.86; 95% CI 1.08–3.18; P for trend = 0.022), MCNP (OR 1.85; 95% CI 1.18–2.91; P for trend = 0.011), MBP (OR 1.68; 95% CI 1.02–2.76; P for trend = 0.041) and MECPP (OR 2.22; 95% CI 1.28–3.86; P for trend = 0.008) in the highest tertile compared to those in the lowest tertile. In the WQS model, the WQS index was significantly positively associated with CVD (OR 1.44; 95% CI 1.04–1.99; *p* value = 0.028). Among the ten phthalates, MCNP showed the highest weight (weighted 0.21). A positive link between phthalate mixture exposure and cardiovascular disease was also demonstrated by the BKMR model. The conditional posterior inclusion probabilities (condPIPs) obtained from the BKMR model indicated that MCNP and MECPP were the primary contributors to the overall effect observed in the group, with condPIP values of 0.581 and 0.508, respectively.

**Conclusion:**

The results indicated that phthalate exposure was linked to an elevated risk of cardiovascular disease and highlighted the need to reduce plastic use among the CKD population.

## 1 Introduction

Since plastics were invented in 1909 (1), they have been ubiquitous in our daily lives as the primary synthetic materials, such as bottled water, food packaging bags, cosmetics, and paints. Plastic particles with a diameter smaller than 5 mm are known as microplastics (MPs), which are degradation products of plastics (2). MPs enter the human body via ingestion, inhalation, and dermal contact (3). Human tissues and body fluids have shown the presence of microplastics, including arteries (4), lungs (5), liver (6), placenta (7), blood (8), urine (9) and breast milk (10).

Phthalates are plasticizers that increase the flexibility and elasticity of plastics. Phthalates play a significant role in the plastic production process. Exposure to phthalates is associated with many health problems, such as depression (11), cancer (12), obesity (13), and diabetes (14). In recent years, several studies have indicated that phthalate exposure is associated with cardiovascular disease. Marfella et al.’s (15) study showed that patients with microplastics detected in carotid artery plaques had a higher risk of developing cardiovascular disease than those who did not have these substances detected. Workers in a chemical plant who were chronically exposed to plastics-related pollution exhibited a higher susceptibility to cardiovascular disease compared to the general population (16). Zhang et al. (17) observed a positive link between urinary phthalates metabolites and serum levels of high-sensitivity cardiac troponin I among the general population. A cross-sectional study in China revealed the positive association between phthalate exposure and CVD in diabetic patients (18). Animal experiments have suggested that MPs cause toxicity to the cardiovascular system, such as reduced heart function, abnormal heartbeat, pericardial edema, and fibrosis of the myocardial tissue (19–21).

CKD is a public health issue, affecting approximately 10% of adults worldwide (22). Individuals with CKD face a greater risk of developing cardiovascular diseases. Cardiovascular disease ranks as the top cause of death for those with CKD (22). In CKD stages 4 to 5, cardiovascular mortality constitutes 40 to 50% of patient deaths (23). Traditional risk factors for cardiovascular disease include hypertension, diabetes, obesity, hyperlipidemia, smoking, high levels of sodium intake, and low physical activity (24). The phthalate is a new risk factor for CVD that has been discovered in recent years. Renal excretion is one of the main ways to remove phthalates from the body (25). CKD patients have impaired renal function, making it easier for phthalates to accumulate in the body. So far, no studies have explored the link between phthalate exposure and CVD in individuals with CKD. In addition, most of the previous studies have only assessed the association between individual phthalate exposure and CVD without considering the effects of mixed exposures.

Therefore, we utilized three statistical models in this study to investigate the effects of exposure to phthalates and the prevalence of CVD in the CKD population, both individually and collectively.

## 2 Methods

### 2.1 Study design and population

**Figure 1:**
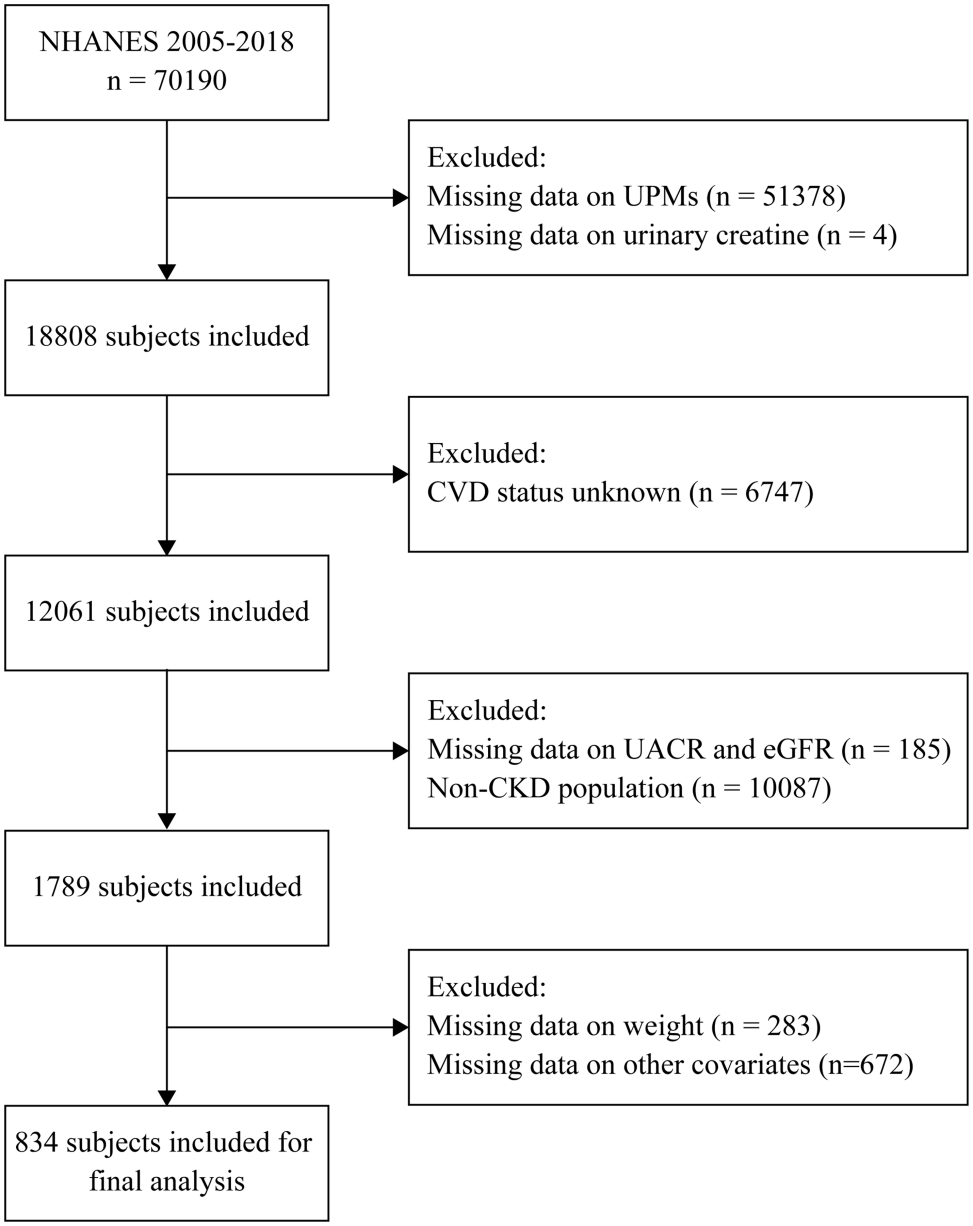
Flowchart of the sample selection from NHANES 2005–2018.

The National Health and Nutrition Examination Survey (NHANES) is a cross-sectional study, which was conducted by the National Center for Health Statistics (NCHS) of the Centers for Disease Control and Prevention (CDC) in the United States. The NHANES survey was designed to assess the health and nutritional status of the U.S. general population. From 1999 to 2018, a sample of around 5,000 individuals from across the nation was examined annually. All individuals involved provided their informed consent in writing. The design, methods, and data of the survey are available on the internet at https://wwwn.cdc.gov/nchs/nhanes/. The data from continuous NHANES 2005–2018 was analyzed, and 70,190 participants were enrolled. We excluded subjects who had missing data on urinary phthalate metabolites, urinary creatinine, CVD outcomes, urinary albumin creatinine ratio, eGFR, sample weights, and other covariates. The study ultimately involved 834 CKD participants (Figure 1).

### 2.2 Urinary phthalate metabolites assessment

Throughout NHANES 2005–2018, twelve metabolites of urinary phthalates were consistently measured. In this study, ten UPMs with concentrations above the lower limit of detection (LLOD) in over 60% of participants were examined (Supplementary Table S1). Values under the LLOD were replaced by the LLOD/ $\sqrt{2}$ . The high-performance liquid chromatography-electrospray ionization-tandem mass spectrometry (HPLC-ESI-MS) was utilized to quantitatively detect urinary phthalate metabolites. The NHANES website contains detailed information on laboratory procedures.

### 2.3 Assessment of CVD outcomes

The assessment of CVD involved a questionnaire where individuals aged 20 years or older reported their medical history. All individuals involved were questioned as follows: “Has a doctor or other health professional ever told you that you had congestive heart failure/coronary heart disease/angina/heart attack/a stroke?” If they responded “Yes” to any of the above questions, they would be considered as having CVD.

### 2.4 Definition of CKD

To generate the estimated glomerular filtration rate (eGFR), the Chronic Kidney Disease-Epidemiology Collaboration (CKD-EPI) formula was applied (26). CKD was defined as either of the following presents for a minimum of 3 months: urinary albumin creatinine ratio ≥ 30 mg/g or eGFR < 60 mL/min/1.73m^2^ (27).

### 2.5 Covariates

We collected information on demographic characteristics (age, gender, race/ethnicity), socio-behavioral characteristics (poverty income ratio), lifestyle (smoking status and BMI), medical history (hypertension and diabetes), and laboratory data (total cholesterol, serum creatinine, and urine creatinine). PIR is the ratio of family income to poverty. A PIR lower than 1 signifies poverty. People were considered smokers if they had smoked at least 100 cigarettes over the course of their life. Hypertension was identified according to a history of diagnosis by a doctor as reported by the individual. Diabetes was recognized by any of the following conditions: (1) HbA1c ≥ 6.5% or fasting glucose ≥ 126 mg/dL; (2) self-reported diagnosis of diabetes; (3) self-reported use of insulin or other diabetes medications. BMI was calculated by dividing a person’s weight in kilograms by their height in meters squared.

### 2.6 Statistical analysis

Urinary phthalate metabolites were corrected for dilution differences in spot urine samples by adjusting for urine creatinine. As the distributions of urinary phthalate metabolites were skewed, the data was ln-transformed to better align with a normal distribution. Continuous variables, including age, PIR, BMI, eGFR, and total cholesterol, were expressed as mean and standard deviation (SD). Group differences of continuous variables were compared using the survey design-based Kruskal-Wallis test. Categorical variables, including gender, race/ethnicity, smoking status, hypertension, and diabetes, were shown in the form of numbers and percentages. Group differences of categorical variables were compared using the survey design-based Rao-Scott Chi-square test.

#### 2.6.1 Generalized linear regression model

Multivariable logistic regression models were utilized to estimate odds ratios (ORs) and 95% confidence intervals (CIs) for the association between the prevalence of cardiovascular disease and individual phthalates. Each phthalate was divided into tertiles, and the lowest tertile (T1) was regarded as the reference group. The median phthalate levels within each tertile were treated as a continuous variable for linear trend analyses. We also conducted logistic regression models with phthalate concentrations, which were treated as continuous variables. All multivariable logistic regression models were adjusted for age, gender, race/ethnicity, PIR, BMI, smoking status, hypertension, diabetes, eGFR, and total cholesterol.

#### 2.6.2 Weighted quantile sum (WQS) regression model

We applied the WQS regression model to assess the effect of phthalate mixture exposure on cardiovascular disease. This method took all the phthalates into consideration and calculated the WQS index, which reflected the impact of all phthalates on the outcome. The model restricted all phthalates to exert the same directional effect on cardiovascular disease. How each phthalate contributed to the total impact was assessed by assigning the weight of each phthalate to the WQS index using the model. Random division of the data resulted in two parts, with 40% designated for training and 60% for validation. The model calculated the average weight for each phthalate after bootstrapping 10,000 times.

#### 2.6.3 Bayesian kernel machine regression (BKMR) model

Considering potential nonlinear and non-addictive relationships between phthalates and CVD, we applied the BKMR model to examine the health impacts of exposure to phthalates. The BKMR model represents a novel method developed to estimate the overall and single-exposure impacts of combinations of multiple pollutants on health (27). It can also identify interactions among individual components of the mixture. This approach employs a kernel function to flexibly estimate the multivariable exposure response function, accommodating nonlinear and non-additive effects. A method for selecting variables hierarchically was created to tackle multicollinearity by grouping highly correlated exposures. In addition, this method makes it possible to visualize functions of exposure and response in high dimensions. Given that the outcome (with or without CVD) was binary in this study, we implemented a probit BKMR regression model. Ten thousand of iterations were used to fit the model. The use of a Gaussian forecasting process method reduced the runtime and ensured accuracy. Posterior inclusion probabilities (PIPs) were generated through the variable selection. The importance of each variable is shown by PIPs, which have values from 0 to 1.

R software (version 4.4.0) was utilized for all the statistical analyses, with a two-sided *p*-value threshold < 0.05 considered statistically significant.

## 3 Results

### 3.1 Baseline characteristics of participants

In the final analysis, there were 834 participants, and 54% of them were females. Participants had a weighted average age of 63.3 years. Race/ethnicity distribution differed between groups with and without CVD. In contrast to the non-CVD group, the CVD group consisted of older individuals, more males, and a larger percentage of people with hypertension, diabetes, and smoking habits. In the CVD group, BMI was higher, while PIR and eGFR were lower. Higher total cholesterol was observed in the non-CVD group, which might be attributed to the CVD group taking lipid-lowering drugs. Table 1 displayed the detailed baseline characteristics of this study.

**Table 1:** Characteristics of the study participants.

Variables	Overall (*n* = 834)	CVD		*p* value
		Yes (*n* = 271)	No (*n* = 563)	
Age (years)	63.3 (15.7)	71.1 (9.6)	60.4 (16.5)	<0.001
Gender				0.023
Female	404 (54%)	105 (46%)	299 (57%)	
Male	430 (46%)	166 (54%)	264 (43%)	
Race/ethnicity				0.035
Mexican American	120 (7.6%)	30 (4.5%)	90 (8.8%)	
Other Hispanic	61 (3.9%)	16 (2.6%)	45 (4.4%)	
Non-Hispanic White	404 (69.2%)	154 (76.2%)	250 (66.6%)	
Non-Hispanic Black	185 (12.4%)	59 (12.0%)	126 (12.5%)	
Other races	64 (6.9%)	12 (4.7%)	52 (7.7%)	
Family PIR	2.82 (1.65)	2.54 (1.57)	2.93 (1.66)	0.032
BMI (kg/m^2^)	30.95 (7.68)	31.93 (7.35)	30.59 (7.78)	0.080
Smoking				<0.001
Yes	418 (47%)	165 (60%)	253 (42%)	
No	416 (53%)	106 (40%)	310 (58%)	
Hypertension				<0.001
Yes	563 (67%)	218 (83%)	345 (61%)	
No	271 (33%)	53 (17%)	218 (39%)	
Diabetes				<0.001
Yes	486 (51%)	189 (68%)	297 (44%)	
No	348(49%)	82 (32%)	266 (56%)	
eGFR (mL/min/1.73m^2^)	70.00 (29.08)	54.87 (21.20)	75.67 (29.62)	<0.001
TC (mg/dL)	183.97 (42.63)	169.75 (43.16)	189.31 (41.22)	<0.001

Continuous variables are presented as mean (SD). Categorical variables are presented as unweighted frequency counts (weighted percentages). PIR, poverty income ratio; BMI, body mass index; eGFR, estimated glomerular filtration; TC, total cholesterol.

### 3.2 Measurement and correlations of urinary phthalate metabolites

**Figure 2:**
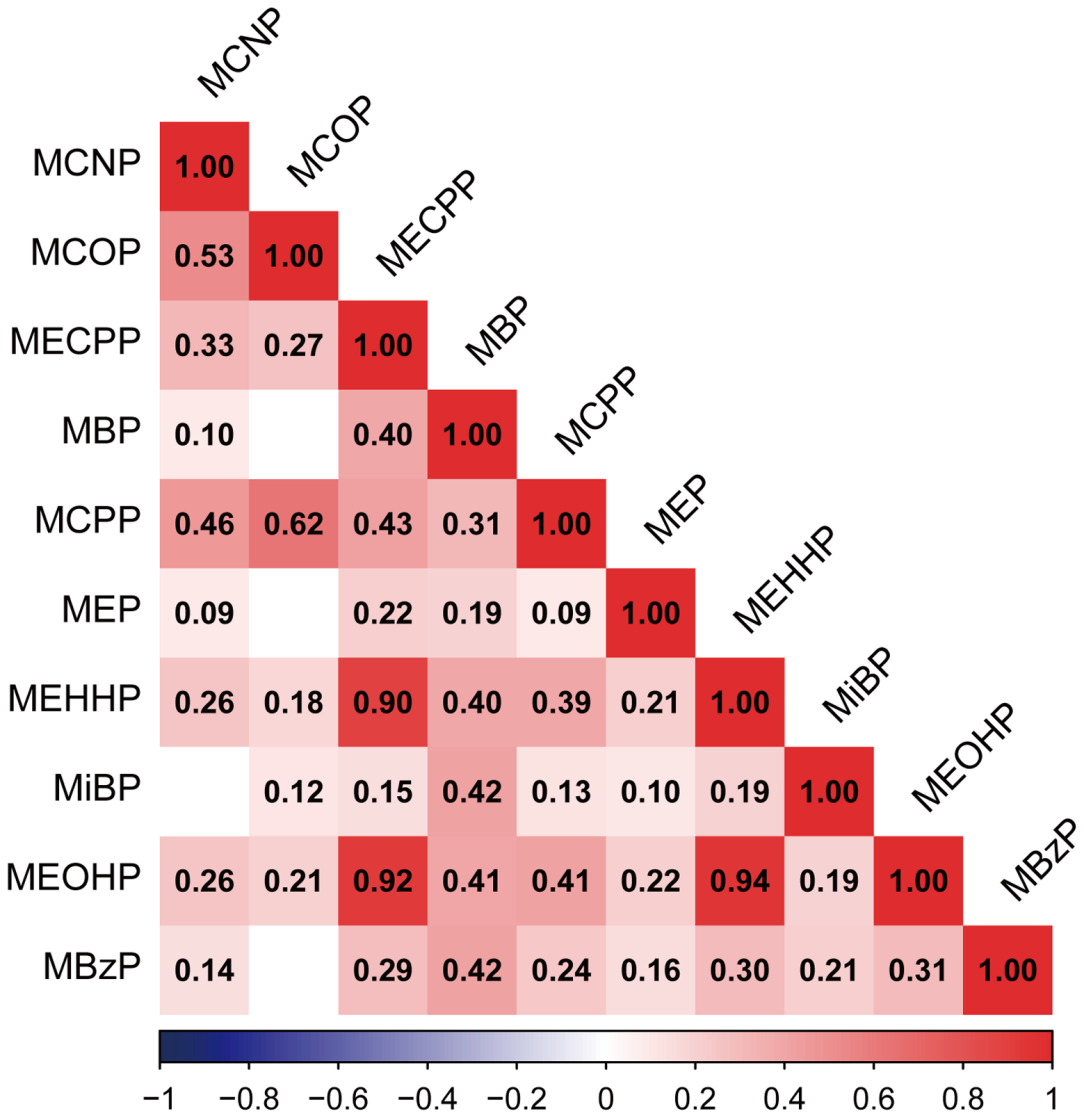
Pearson correlations among urinary concentrations of ten phthalates in the population (*N* = 834). All of the correlations showed statistical significance (*p* < 0.05), except those of MBP and MCOP (*p* = 0.28), MEP and MCOP (*p* = 0.31), MiBP and MCNP (*p* = 0.3), and MBzP and MCOP (*p* = 0.06).

The detection frequency and distribution of phthalates were shown in Supplementary Table S1. There were ten phthalates with a detection frequency above 60%. We found significant positive correlations among 10 phthalates (*r* ranging from 0.09 to 0.94). There were strong correlations between MEOHP and MEHHP (*r* = 0.94), MEOHP and MECPP (*r* = 0.92), and MEHHP and MECPP (*r* = 0.90) (Figure 2).

### 3.3 Generalized linear regression model to evaluate the association between phthalates and CVD

We applied multivariable logistic regression to assess the effect of each phthalate on CVD. In the multivariable logistic regression model, we adjusted for covariates, including age, gender, race/ethnicity, PIR, BMI, smoking status, hypertension, diabetes, GFR, and total cholesterol. Table 2 indicated the association between phthalate exposures and the prevalence of CVD. After adjusting for potential covariates, compared to the lowest tertile, MiBP (OR 1.86; 95% CI 1.08–3.18; P for trend = 0.022), MCNP (OR 1.85; 95% CI 1.18–2.91; P for trend = 0.011), MBP (OR 1.68; 95% CI 1.02–2.76; P for trend = 0.041) and MECPP (OR 2.22; 95% CI 1.28–3.86; P for trend = 0.008) in the highest tertile were independently related to the elevated prevalence of CVD. No significant associations were found between the other urinary phthalate metabolites and CVD. Supplementary Table S2 displayed the relationship between each phthalate and CVD by considering the concentration of each phthalate as a continuous variable. MiBP (OR 1.30; 95% CI 1.01–1.68; *p* = 0.041), MCNP (OR 1.26; 95% CI 1.03–1.53; *p* = 0.027) and MBP (OR 1.34; 95% CI 1.06–1.71; *p* = 0.017) were significantly associated with CVD.

**Table 2:** The association between individual phthalate concentration and the prevalence of CVD.

Variables	OR (95% CI)	*p* value	P for trend
MEP			
T1	Ref		
T2	1.08 (0.61, 1.92)	0.779	
T3	0.98 (0.63, 1.54)	0.944	0.915
MEHHP			
T1	Ref		
T2	0.70 (0.41, 1.20)	0.192	
T3	1.34 (0.77, 2.35)	0.295	0.207
MiBP			
T1	Ref		
T2	1.59 (0.93, 2.75)	0.092	
T3	1.86 (1.08, 3.18)	0.025	0.022
MEOHP			
T1	Ref		
T2	1.53 (0.89, 2.63)	0.123	
T3	1.67 (0.96, 2.91)	0.068	0.076
MBzP			
T1	Ref		
T2	1.19 (0.65, 2.17)	0.577	
T3	1.32 (0.78, 2.25)	0.295	0.293
MCNP			
T1	Ref		
T2	1.43 (0.89, 2.28)	0.134	
T3	1.85 (1.18, 2.91)	0.008	0.011
MCOP			
T1	Ref		
T2	1.16 (0.65, 2.05)	0.617	
T3	1.39 (0.83, 2.35)	0.210	0.205
MECPP			
T1	Ref		
T2	1.90 (1.10, 3.26)	0.021	
T3	2.22 (1.28, 3.86)	0.005	0.008
MBP			
T1	Ref		
T2	1.20 (0.69, 2.10)	0.504	
T3	1.68 (1.02, 2.76)	0.042	0.041
MCPP			
T1	Ref		
T2	0.82 (0.51, 1.32)	0.414	
T3	1.44 (0.93, 2.23)	0.104	0.081

By comparing the second and third tertiles of each phthalate to the first, odds ratios (ORs) were calculated. The models were adjusted for age, gender, race/ethnicity, PIR, BMI, smoking status, hypertension, diabetes, total cholesterol, and eGFR.

A subgroup analysis was also carried out to compare the association among different renal functions. MECPP was associated with a higher prevalence of CVD in participants with eGFR ranging from 60 to 90 mL/min/1.73m^2^ (OR 3.31; 95% CI 1.62–6.79; P for interaction = 0.029). MBP was associated with a higher prevalence of CVD in participants with eGFR lower than 60 mL/min/1.73m^2^ (OR 1.59; 95% CI 1.12–2.25; P for interaction = 0.036) (Table 3). No significant interaction was found for the other phthalates.

**Table 3:** The association between urinary phthalate metabolites and the prevalence of cardiovascular disease among CKD patients with different renal functions.

Phthalates	>90 mL/min/1.73m^2^ (*n* = 221)	60–90 mL/min/1.73m^2^ (*n* = 156)	<60 mL/min/1.73m^2^ (*n* = 457)	P for interaction
MEP	0.99 (0.62, 1.59)	0.93 (0.72, 1.20)	1.08 (0.89, 1.32)	0.850
MEHHP	0.91 (0.41, 2.05)	1.68 (0.88, 3.20)	1.13 (0.88, 1.46)	0.852
MiBP	1.03 (0.34, 3.15)	1.96 (1.15, 3.35)	1.20 (0.89, 1.62)	0.907
MEOHP	0.93 (0.39, 2.21)	2.50 (1.29, 4.84)	1.11 (0.86, 1.45)	0.477
MBzP	1.19 (0.65, 2.16)	1.16 (0.72, 1.88)	1.12 (0.86, 1.46)	0.577
MCNP	1.85 (0.98, 3.51)	1.23 (0.77, 1.96)	1.19 (0.89, 1.58)	0.186
MCOP	1.46 (0.80, 2.65)	1.63 (1.00, 2.67)	1.00 (0.82, 1.23)	0.076
MECPP	1.44 (0.63, 3.31)	3.31 (1.62, 6.79)	1.12 (0.85, 1.46)	0.029
MBP	0.71 (0.27, 1.85)	0.99 (0.43, 2.26)	1.59 (1.12, 2.25)	0.036
MCPP	1.00 (0.60, 1.66)	1.29 (0.73, 2.28)	1.05 (0.87, 1.26)	0.995

The models were adjusted for age, gender, race/ethnicity, PIR, BMI, smoking status, hypertension, diabetes, total cholesterol, and eGFR.

### 3.4 WQS regression model to evaluate the association between phthalates and CVD

**Figure 3:**
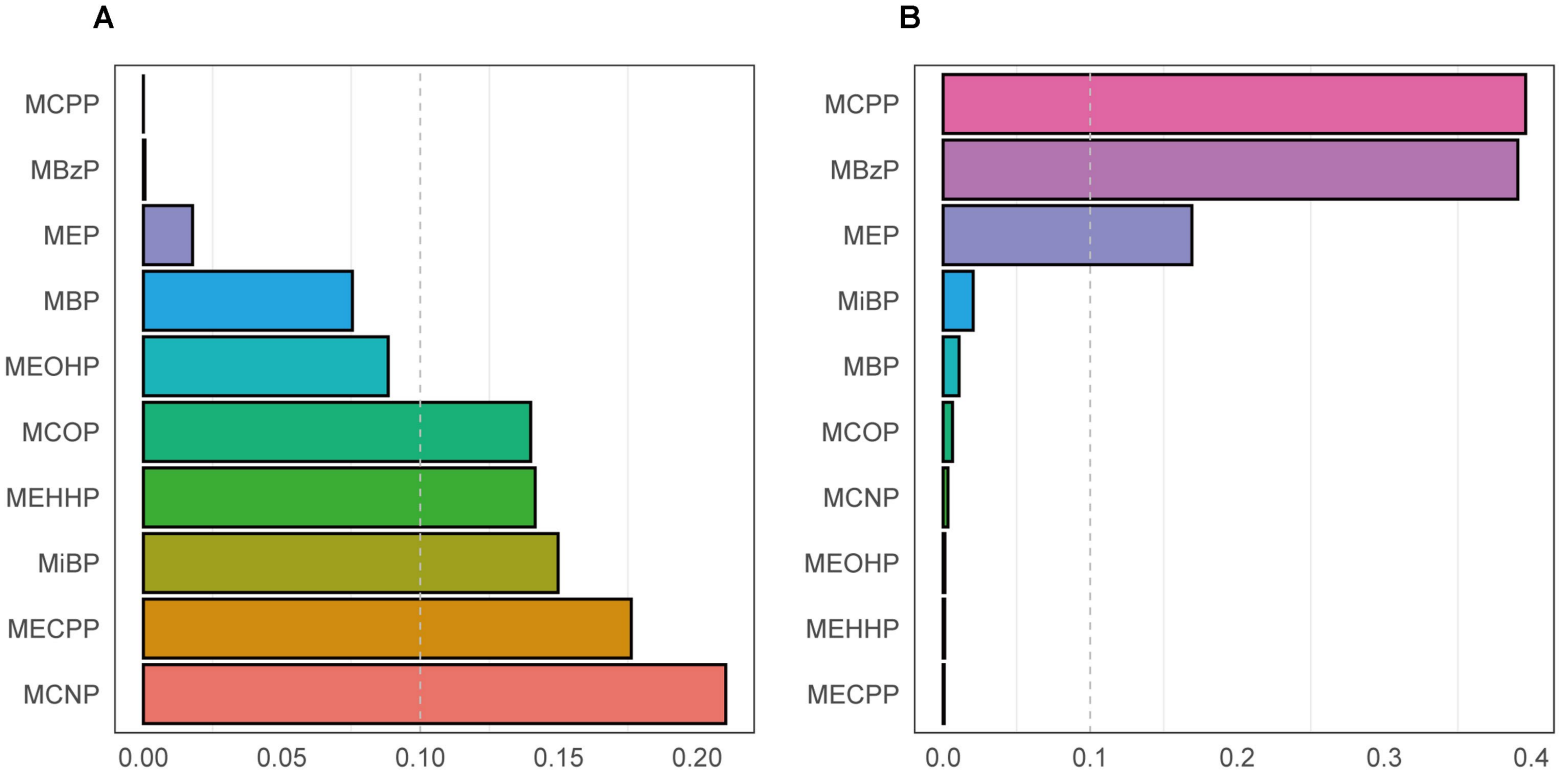
The weight of each phthalate for the WQS index in a positive WQS regression model **(A)** and a negative WQS regression model **(B)**. Models were adjusted for age, gender, race/ethnicity, PIR, BMI, smoking status, hypertension, diabetes, total cholesterol, and eGFR.

We utilized the WQS regression model to assess the effect of phthalate mixture exposure on CVD. As presented in Table 4, after adjustment for all covariates, the WQS index was significantly associated with CVD, with an odds ratio of 1.44 (95% CI 1.04–1.99). The weight of each phthalate for the WQS index was reported in Figure 3A. Among the ten phthalates, MCNP was weighted the most at 0.21, with MECPP and MiBP having weights of 0.18 and 0.15, respectively.

**Table 4:** Association between WQS regression index and cardiovascular disease (*N* = 834), NHANES, 2005–2018.

Outcomes	OR	95% CI	*p* value
CVD			
A positive model	1.44	(1.04, 1.99)	0.028
A negative model	1.30	(0.95, 1.77)	0.102

OR estimates reflect the odds ratios of CVD with a one-quartile increase in the WQS index. Models were adjusted for age, gender, race/ethnicity, PIR, BMI, smoking status, hypertension, diabetes, total cholesterol, and eGFR.

Considering the WQS model hypothesized that all phthalates exerted the same directional effect on cardiovascular disease, we also fitted the negative WQS model to examine whether the WQS index was negatively associated with CVD. Each phthalate’s weight was displayed in Figure 3B. No significant association was found in this model (Table 4).

### 3.5 BKMR model to evaluate the association between phthalates and CVD

We employed the BKMR model to determine the combined impact of phthalate exposure on CVD. Table 5 provided a summary of the inclusion probabilities for each phthalate. The primary influence of the entire group was driven by MCNP and MECPP (condPIP = 0.581 and 0.508, respectively), while the condPIPs of others were lower than 0.5.

**Table 5:** CondPIP in BKMR model in NHANES 2005–2018 (*N* = 834).

Phthalates	CondPIP
MCNP	0.581
MECPP	0.508
MEP	0.481
MiBP	0.471
MEHHP	0.470
MEOHP	0.458
MCOP	0.458
MCPP	0.447
MBzP	0.430
MBP	0.383

The BKMR model was adjusted for age, gender, race/ethnicity, PIR, BMI, smoking status, hypertension, diabetes, total cholesterol, and eGFR. CondPIP, conditional posterior inclusion probability.

**Figure 4:**
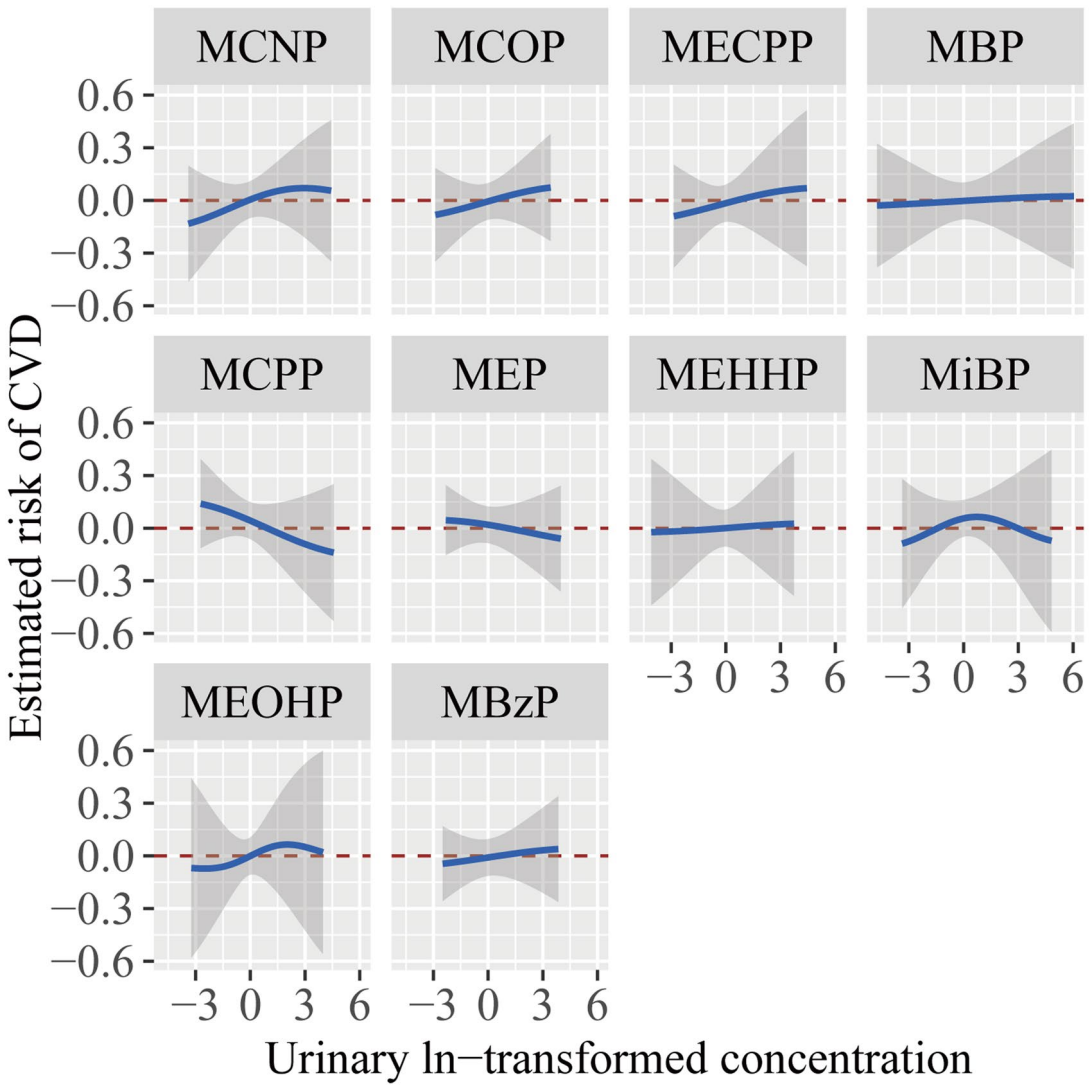
The estimated risk and 95% CI for each phthalate exposure with CVD while setting other phthalates to their median concentrations. The models were adjusted for age, gender, race/ethnicity, PIR, BMI, smoking status, hypertension, diabetes, total cholesterol, and eGFR.

Figure 4 illustrated the association of each phthalate with CVD, while the remaining nine phthalates were maintained at their median concentrations. MCNP, MCOP, MECPP, and MBzP showed a positive relationship with CVD, whereas MBP, MCPP, MEP, and MEHHP exhibited a flat or inverse correlation. MiBP and MEOHP exhibited increasing association with CVD in the low concentration and inverse association in the high concentration. However, considering the 95% confidence intervals included the reference line of 0, these results were not statistically significant.

**Figure 5:**
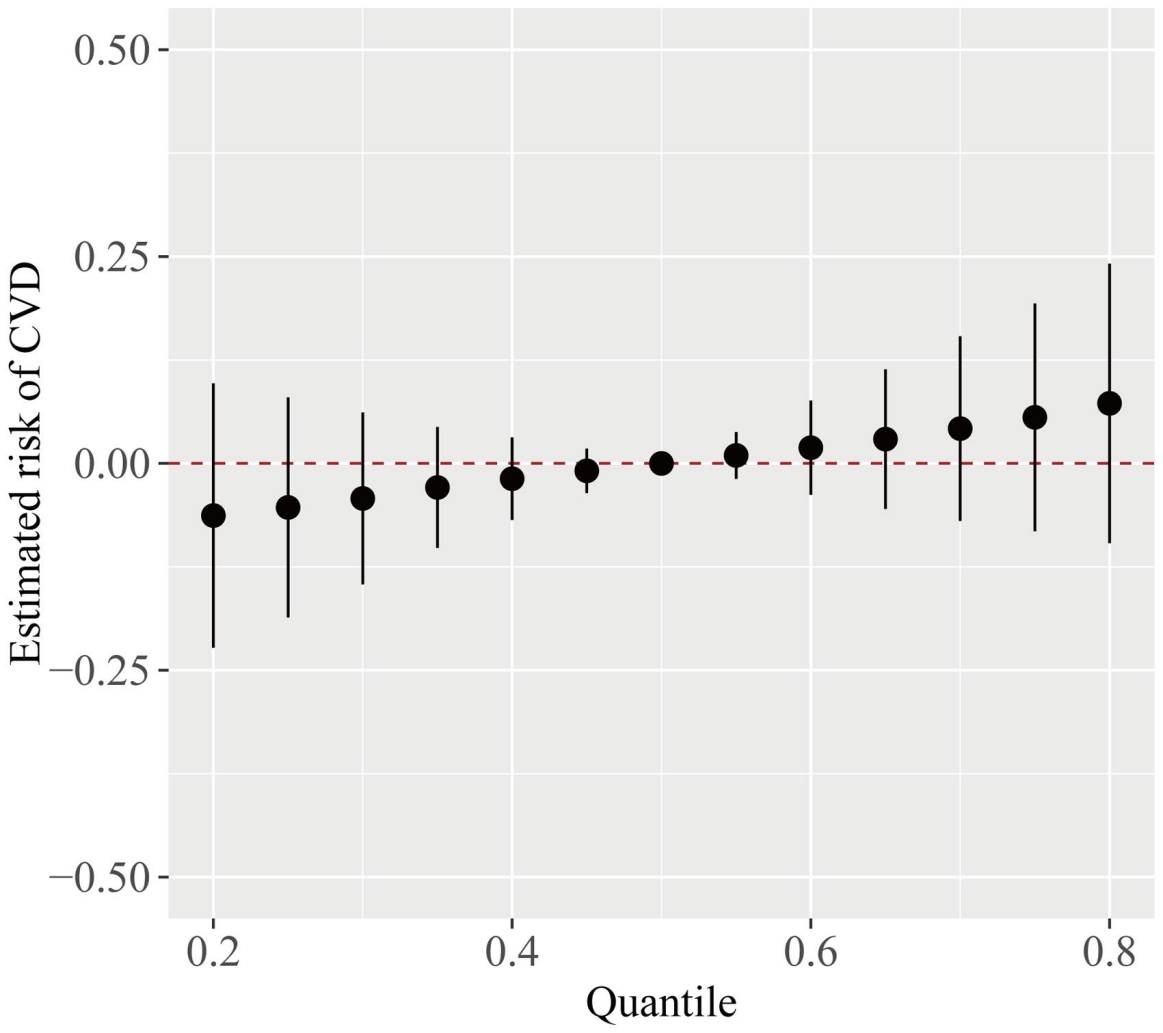
The overall CVD risk (95% CI) from phthalate exposure when comparing different phthalates at various percentiles to their median level. The model was adjusted for age, gender, race/ethnicity, PIR, BMI, smoking status, hypertension, diabetes, total cholesterol, and eGFR.

Figure 5 displayed the general link between exposure to the phthalate mixture and cardiovascular disease. Despite the lack of a statistically significant difference in the model (95% CI including zero), there was a positive trend between the phthalate mixture exposure and CVD. We further investigated the interactions between phthalates. As shown in Supplementary Figure S1, when the association between exposure 1 and CVD was affected by levels of exposure 2, the curves intersected, which indicated potential interactions existed between phthalates.

## 4 Discussion

In this cross-sectional study involving 834 CKD participants, we applied three statistical models to analyze the relationship between phthalates and cardiovascular diseases. In the generalized linear regression model, after adjusting for all covariates, we found that MiBP, MCNP, MECPP, and MBP were associated with CVD. In the fully adjusted WQS model, the WQS index was significantly and positively related to CVD. Among the ten phthalates, MCNP showed the highest weight. Pearson correlations indicated that MECPP, MEHHP, and MEOHP are highly correlated. The BKMR model also demonstrated a positive trend between phthalate mixture exposure and CVD. The condPIPs derived from the BKMR model showed that the main effect on the entire group was primarily due to MCNP.

The findings of our study partially aligned with several previous studies. In a cross-sectional study, Zhu et al. (28) reported that MiBP and MBzP were associated with the increased prevalence of CVD among the general adult population. Additionally, Su et al.’s (29) study showed an association between urinary phthalates metabolites (MEHP, MnBP, and MiBP) and coronary heart disease. Another survey by Shiue indicated that MBP was associated with the risk of stroke (30). However, Olsen et al. (31) observed no association between four selected phthalate metabolites (MiBP, MMP, MEP, and MEHP) and coronary risk in an older adult population. It is worth noting that none of the aforementioned studies considered renal function when performing multivariate analyses.

Several studies have indicated that micro-and nanoplastics (MNPs) can be excreted in the urine. In human urine samples, microplastics were detected by Raman microspectroscopy (9). Nanoplastics were rapidly detected in urine after a single exposure, which included tail vein injection, gavage, or pulmonary perfusion in mice (32). These studies revealed that kidney excretion plays a vital role in the elimination of MNPs. CKD patients have declined eGFR levels, making CKD a condition more prone to MNPs accumulation. Furthermore, phthalates may adversely affect the kidneys, forming a vicious circle. An inverse relationship was observed between phthalate metabolites and eGFR, along with a positive relationship with urinary ACR among general adults (33). A cross-sectional clinical study also showed that urinary phthalate metabolites were associated with renal impairment (34). In addition, CKD patients have compromised intestinal epithelial barrier function, making it easier for MNPs to pass through the intestinal barrier and reach the bloodstream (35). In our study, in the subgroup analysis, MECPP was associated with CVD among CKD patients with eGFR 60–90 mL/min/1.73m^2^, while MBP exhibited association with CVD among CKD patients with eGFR < 60 mL/min/1.73m^2^. Differences in this relationship under different kidney function statuses may be explained by a negative association between urinary creatine and renal function. We used urinary creatine to adjust for urinary dilution of phthalates. Urinary creatine levels can be influenced by eGFR, which may introduce a bias (36).

Although the underlying mechanism of cardiovascular toxicity induced by phthalates has not been entirely clarified, several insights have been put forward in recent years. Chen et al.’s (37) study showed that microplastic exposure altered the expression of genes associated with heart development in fish. Sun et al. (21) observed pericardial edema in zebrafish embryos exposed to nanoplastics. The expression of genes involved in the development of atrioventricular heart valves was significantly affected after exposure to nanoplastics in human induced pluripotent stem cells (hiPSCs) (38). These findings imply that MNPs adversely affect early cardiac development. Zhang et al. (39) found that microplastics induced mitochondrial damage and energy metabolism dysfunction via the AMPK-PGC-1α pathway in cardiomyocytes of chickens. Li et al. (40) identified that microplastics triggered oxidative stress and induced cardiomyocyte apoptosis, finally leading to cardiac fibrosis in rats. Wei et al.’s (41) study revealed that the administration of microplastics in rats induced pyroptosis of cardiomyocytes. Moreover, inflammation significantly contributes to the onset of cardiovascular diseases. The expression levels of inflammatory markers in plaque samples were higher in patients in whom MNPs were detected within the plaque than those in whom MNPs were not detected (15). Studies performed *in vitro* and *in vivo* revealed that MNPs triggered cardiomyocyte inflammation responses (42–44). In an observational cohort study, Amdur et al. (45) found that the plasma levels of inflammation biomarkers were independently associated with atherosclerotic vascular disease in CKD patients. Therefore, phthalates may also induce cardiovascular toxicity in CKD patients by aggravating the inflammatory response.

This study has some strengths and limitations. This study is the first to explore the correlation between phthalates and cardiovascular disease among those with CKD. Furthermore, by employing three statistical models, we comprehensively estimated the single exposure, overall exposure, and interactive effects of phthalates on the CVD outcome. This study also comes with a few limitations that should be noted. First, we excluded the population whose urinary phthalates data were missing. This population may include CKD participants, which might lead to a bias in our study. Second, although this study has adjusted for a variety of covariates, there may be other potential confounders that were not considered. Third, the CVD outcome was assessed using a self-reported questionnaire, which may lack accuracy. Finally, this study is cross-sectional and thus cannot conclude the causal link between phthalate exposure and CVD outcome. Hence, to further examine the link between phthalate exposure and cardiovascular disease, prospective and large-scale cohort studies are required.

## 5 Conclusion

Our study concludes that urinary phthalate metabolites are associated with CVD among individuals with CKD, with the greatest influence being from MCNP. These findings indicate that phthalate exposure is a potential risk factor for CVD and highlight the need to reduce phthalate exposure in the CKD population.

## Data Availability

Publicly available datasets were analyzed in this study. This data can be found at: https://wwwn.cdc.gov/nchs/nhanes/.

## Supplementary material

SUPPLEMENTARY FIGURE S1 Association between exposure 1 and CVD when exposure 2 was held at the 10th, 50th, and 90th percentiles (and maintaining the remaining phthalates at their median levels). Models were adjusted for age, gender, race/ethnicity, PIR, BMI, smoking status, hypertension, diabetes, total cholesterol, and eGFR.
