# Supplementary figures and images for: Individual and joint effects of exposure to phthalates and the risk of cardiovascular disease in the chronic kidney disease population: NHANES 2005–2018
Source: Front Public Health. 2025 May 14;13:1579618. doi: 10.3389/fpubh.2025.1579618 (PMC12116607; doi:10.3389/fpubh.2025.1579618)

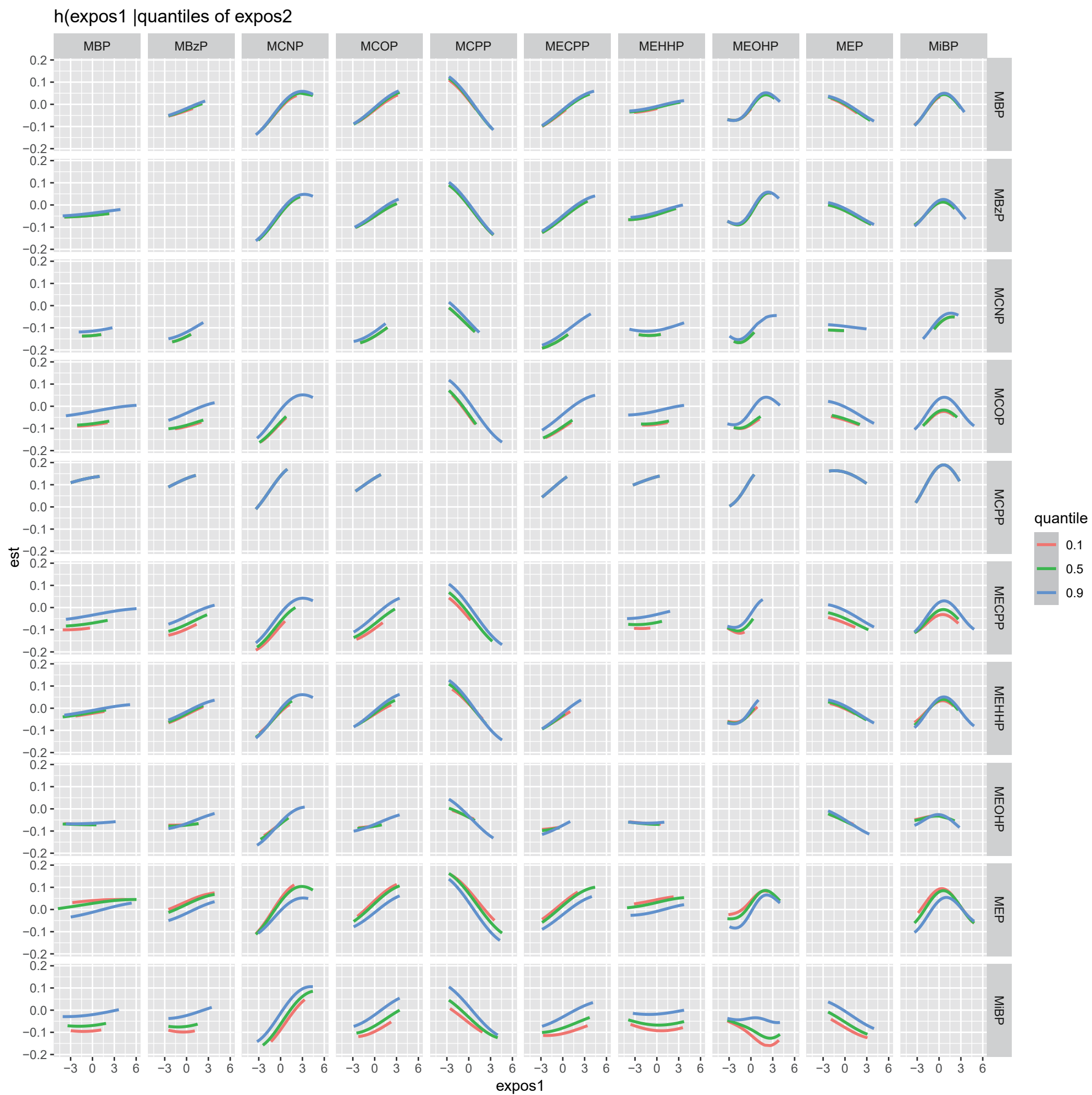

Supplement: SUPPLEMENTARY FIGURE S1 — Association between exposure 1 and CVD when exposure 2 was held at the 10th, 50th, and 90th percentiles (and maintaining the remaining phthalates at their median levels). Models were adjusted for age, gender, race/ethnicity, PIR, BMI, smoking status, hypertension, diabetes, total cholesterol, and eGFR. [file Image_1.pdf]
