# Supplementary material for: Individual and joint effects of exposure to phthalates and the risk of cardiovascular disease in the chronic kidney disease population: NHANES 2005–2018
Source: Front Public Health. 2025 May 14;13:1579618. doi: 10.3389/fpubh.2025.1579618 (PMC12116607; doi:10.3389/fpubh.2025.1579618)
Supplement: Supplementary file 2 [file Data_Sheet_1.docx]

**Supplementary Table S1.**

The detection frequency and distribution of the concentrations of phthalates (N = 834).

| Phthalates | Detection frequency | Min | 25th percentile | Median | 75th percentile | Max |
| --- | --- | --- | --- | --- | --- | --- |
| MCNP | 97.3% | 0.14 | 1.00 | 1.97 | 3.54 | 190.60 |
| MCOP | 98.6% | 0.21 | 3.60 | 7.70 | 19.26 | 674.02 |
| MECPP | 99.9% | 0.28 | 6.61 | 13.3 | 27.06 | 975.35 |
| MBP | 99.5% | 0.28 | 6.03 | 11.90 | 21.50 | 2768.40 |
| MCPP | 88% | 0.14 | 0.80 | 1.62 | 3.66 | 294.50 |
| MEP | 99.9% | 0.85 | 16.90 | 41.60 | 123.11 | 8296.90 |
| MEHHP | 99.5% | 0.21 | 3.90 | 8.17 | 17.34 | 929.50 |
| MEHP | 49.8% | 0.35 | 0.57 | 0.85 | 2.00 | 68.12 |
| MiBP | 96.5% | 0.21 | 3.20 | 6.72 | 13.13 | 304.20 |
| MNP | 19.5% | 0.54 | 0.64 | 0.64 | 0.87 | 185.19 |
| MEOHP | 99.0% | 0.14 | 2.70 | 5.40 | 11.35 | 587.84 |
| MBzP | 97.9% | 0.15 | 1.97 | 4.43 | 10.70 | 450.22 |

Unit: ng/ml

**Supplementary Table S2.**

The relationship between each phthalate and CVD by considering the concentration of each phthalate as a continuous variable.

| Phthalates | OR | 95% CI | *P* value |
| --- | --- | --- | --- |
| MEP | 1.02 | 0.90, 1.17 | 0.711 |
| MEHHP | 1.14 | 0.93, 1.40 | 0.200 |
| MiBP | 1.30 | 1.01, 1.68 | 0.041 |
| MEOHP | 1.17 | 0.94, 1.46 | 0.149 |
| MBzP | 1.12 | 0.92, 1.35 | 0.253 |
| MCNP | 1.26 | 1.03, 1.53 | 0.027 |
| MCOP | 1.16 | 0.99, 1.36 | 0.070 |
| MECPP | 1.25 | 0.99, 1.57 | 0.056 |
| MBP | 1.34 | 1.06, 1.71 | 0.017 |
| MCPP | 1.06 | 0.93, 1.22 | 0.372 |

Models were adjusted for age, gender, race/ethnicity, PIR, BMI, smoking status, hypertension, diabetes, total cholesterol, and eGFR.
